# “A tool to support, not replace”: patient and general practitioner perceptions of digital decision support tools for back pain

**DOI:** 10.1093/fampra/cmaf098

**Published:** 2025-12-31

**Authors:** Avi Goodman, Aidan G Cashin, Isha Mishra, Mia Ryan, Jennifer McBride, Steven Marsh, Tianle Xie, Gustavo Batista, Oyungerel Byambasuren, James H McAuley, Rodrigo R N Rizzo

**Affiliations:** School of Health Sciences, University of New South Wales, Sydney, NSW 2052, Australia; Centre for Pain IMPACT, Neuroscience Research Australia, Sydney, NSW 2031, Australia; School of Health Sciences, University of New South Wales, Sydney, NSW 2052, Australia; Centre for Pain IMPACT, Neuroscience Research Australia, Sydney, NSW 2031, Australia; School of Health Sciences, University of New South Wales, Sydney, NSW 2052, Australia; Centre for Pain IMPACT, Neuroscience Research Australia, Sydney, NSW 2031, Australia; School of Health Sciences, University of New South Wales, Sydney, NSW 2052, Australia; Centre for Pain IMPACT, Neuroscience Research Australia, Sydney, NSW 2031, Australia; School of Health Sciences, University of New South Wales, Sydney, NSW 2052, Australia; Centre for Pain IMPACT, Neuroscience Research Australia, Sydney, NSW 2031, Australia; Centre for Pain IMPACT, Neuroscience Research Australia, Sydney, NSW 2031, Australia; School of Computer Science and Engineering, University of New South Wales, Sydney, NSW 2052, Australia; Institute for Evidence-Based Healthcare, Bond University, Robina, QLD 4226, Australia; School of Health Sciences, University of New South Wales, Sydney, NSW 2052, Australia; Centre for Pain IMPACT, Neuroscience Research Australia, Sydney, NSW 2031, Australia; School of Health Sciences, University of New South Wales, Sydney, NSW 2052, Australia; Centre for Pain IMPACT, Neuroscience Research Australia, Sydney, NSW 2031, Australia

**Keywords:** primary health care, pain management, qualitative research, digital health, back pain

## Abstract

**Background:**

Back pain is the leading musculoskeletal reason for visits in general practice. Time constraints on consultations affect diagnostic decisions and the delivery of evidence-based care. This study explored patient and general practitioner (GP) perspectives on digital tools to support decision-making in back pain management.

**Methods:**

We conducted separate focus groups between June and August 2024 with people experiencing back pain and with registered Australian GPs. We analyzed data using thematic analysis with an inductive approach.

**Results:**

We interviewed 23 participants: 13 with back pain and 10 GPs. Patients appreciated digital decision-support tools for increasing knowledge and clarifying persistent questions after consultations. GPs, in contrast, emphasized red flag screening, symptom monitoring, and time savings. Shared concerns included information trustworthiness, lack of personalization, and cost, while facilitators included integration into practice management systems and GP endorsement.

**Conclusions:**

Our findings highlight opportunities to integrate digital decision-support tools at multiple points in GPs’ workflows—before, during, and after consultations—to address the needs of both patients with back pain and GPs. When used before consultations, such tools can help patients prepare by increasing their knowledge, supporting more productive discussions, informing decisions about whether a visit is necessary, and assisting GPs in identifying potential red flags. During consultations, the tools can provide clinicians with updates on current evidence and supply educational resources or prescriptions, particularly for evidence-based lifestyle interventions. After consultations, they can support follow-up by monitoring the patient's condition and addressing any persistent questions that may arise.

Key Messages

- Digital decision-support tools have a role in back pain care.
- The tools are viewed as a complement to GP care, not a replacement.
- They can be used before, during or after the consultation.
- The tools can guide care decisions and support more productive interactions.
- Integrating tools into existing GP workflows can support implementation.

## Background

Back pain constitutes a substantial burden for individuals and society, and for the past three decades, it has remained the leading cause of years lived with disability worldwide [1]. The condition generates significant healthcare costs, with expenses for consultations, diagnostic procedures, treatments, and hospital admissions estimated at €2.3 to €2.6 billion annually [2].

Clinical practice guidelines offer recommendations for managing back pain in general practice [3]. The current recommendation involves assessing patients, screening for specific causes, providing referral when needed, and, for *most* individuals, offering advice and information, selecting appropriate non-drug treatments, and, for *some*, prescribing drug treatments for acute or chronic back pain [4]. Treatment decisions should reflect shared discussions that consider the evidence, patient preferences, and contextual factors [5].

However, implementing these recommendations remains challenging [6]. Patients often arrive with misconceptions about back pain, multisite pain, and comorbidities [7], which can be difficult to address within a single consultation [8], and within the constraints of limited consultation time [9, 10].

The use of decision-support tools, accessed by patients or health professionals via smartphones, email, or computers, has been proposed to support evidence-based care [11, 12]. Examples include digital fact sheets, webpages, search engines (e.g. Google), and conversational agents that patients can access themselves or that are directed by health professionals. These tools might inform management and provide education, offering convenient, fast access to evidence-based information in clinical practice.

Although the use and delivery of digital materials in the general practice context are growing, the perspectives of individuals with back pain and health professionals on digital decision aids for back pain management are unknown. To address this, this study aims to understand the perspectives of individuals with back pain and GPs on digital decision support tools for back pain management.

## Methods

### Study design and ethical approval

We conducted a qualitative study using focus groups [13]. The protocol was prospectively registered on the Open Science Framework (https://doi.org/10.17605/OSF.IO/M5TFJ). The study followed the Declaration of Helsinki, received approval from the University of New South Wales Research Ethics Committee (iRECS6241), and was reported in line with the Consolidated Criteria for Reporting Qualitative Research (see Supplementary Data) [14].

### Reflexivity and assumptions

The authors include a medical doctor (O.B.), a physiotherapist (R.R.N.R.), a psychologist (J.H.M.), computer scientists (G.B. and T.X.), exercise physiologists (A.G., I.M., M.R., and A.C.), and people with lived experience of back pain (J.M. and S.M.). The study was oriented by relativism (i.e. the belief that reality is subjective) and interpretive orientations (i.e. acknowledging that contextual interactions construct knowledge) [15, 16]. R.R.N.R., a male physiotherapist-researcher with experience in qualitative studies and A.G., a male honors exercise physiologist student, moderated the focus groups.

### Participants and recruitment

We included individuals with back pain and Australian-accredited GPs. The eligibility criteria and recruitment strategy are shown in Table 1. Purposive sampling ensured diversity in age and gender. The study was advertised online, and interested participants accessed study information, completed eligibility and consent forms via REDCap, and were contacted by a researcher (A.G.) to schedule focus groups. Participation was reimbursed with AUS$40 gift cards for individuals with back pain and AUS$180 for GPs.

**Table 1:** Eligibility criteria and recruitment strategy.

Group	Participant characteristics	Recruitment strategy
Individuals with back pain	Adults (18 years or older) experiencing back pain of any duration Participants with low back pain living in Australia Participants who understand and speak English Access the internet and a device that can operate Microsoft Teams	Advertised via social media Emailed existing lists of individuals who had expressed interest in participating in future studies at Neuroscience Research Australia (NeuRA)
General Practitioners	Working as an accredited general practitioner in Australia Having provided care to at least five patients with low back pain in the past year Access the internet and a device that can operate Microsoft Teams	External recruitment company (TKW) using general practitioners’ networks. Snowballing approach (recommendations and referrals from participants or professional networks)

The research team determined the number of participants when a sufficient understanding of the phenomenon to inform clinical practice was achieved through data collection, analysis, and discussions [17]. A.G and R.R.N.R completed data collection and analysis concurrently. Based on relevant published research, a minimum of 10 individuals with back pain and 10 GPs were planned to be included [18].

### Data collection and analysis

We collected qualitative data through one-hour online focus groups on Microsoft Teams, held separately with individuals with back pain and GPs (3–6 participants per group). A.G. and R.R.N.R., with revisions from co-authors (including researchers, clinicians, and individuals with lived experience), developed the interview guides based on the Theoretical Domains Framework [19] (see Supplementary Data).

We defined digital decision support tools or decision aids as any digital material or tool accessed by patients or health professionals (e.g. smartphones, email, computers) to support decision-making. Examples might include fact sheets, webpages, search engines, and conversational agents that patients or health professionals can access at any point in the consultation (before, during, or after) to expand on information and facilitate decision-making for back pain management [20]. A.G. and R.R.N.R. conducted the focus groups. A.G. introduced the study, the participants (i.e. their names, locations, and roles in the focus group), and provided the ground rules for the focus group, then initiated the discussion with guiding questions. R.R.N.R provided technological support, took notes, and conducted complementary queries and summaries.

We summarized the demographic data descriptively. We analyzed the qualitative data inductively using Reflexive Thematic Analysis [21, 22]. Two analysts (A.G. and R.R.N.R.) first coded data from one focus group with individuals with back pain and one with GPs independently in NVivo 14, then compared and discussed the codes to ensure consistency. A.G. coded the remaining transcripts and developed preliminary themes, which were refined through discussions with co-authors (R.R.N.R., I.M., M.R., and AC). All authors, including those with lived experience of chronic pain (S.M. and J.M.) and a medical doctor (O.B.), reviewed and refined the themes to ensure a transparent and coherent presentation.

## Results

Overall, 50 individuals with back pain expressed interest in the study. Nineteen did not provide consent, and 18 were unable to participate due to timing conflicts or an inability to contact them. Thirteen individuals with back pain were included. We also recruited 10 GPs—one via snowball sampling and nine through an external recruitment company. Focus group moderators had no prior contact with participants, except for one GP who had participated in a previous study. We conducted a total of five focus groups: three with individuals with back pain and two with GPs. The focus groups were conducted with three to five individuals with back pain, or four to six GPs each.

Most were female (*n* = 8/13, 61%) and identified as White (*n* = 11/13, 85%). The median (range) age was 44 (24–81) years, with a median (range) duration of back pain of 7 (0.2–50) years. Participants reported moderate to severe pain, with a median (range) score of 6 (3–10) on a 0–10 numerical rating scale. GPs were female (5/10, 50%), with a median (range) age of 51 (31–69) years and a median (range) clinical experience of 23.5 (6–31) years. The ethnicity of GPs was predominantly Asian (7/10, 70%). Most GPs were based in NSW (8/10, 80%), with only one (10%) working in rural communities. The demographic and clinical characteristics of participants are presented in Table 2.

**Table 2:** Participants characteristics.

Characteristics	Individuals with back pain (*n* = 13)	General practitioners (*n* = 10)
**Female, *n* (%)**	8 (62%)	5 (50%)
**Age, median (range)**	44 (24–81)	51 (31–69)
**Ethnicity, *n* (%)**		
White	11 (85%)	2 (20%)
Asian	1 (8%)	7 (70%)
Hispanic or Latino	1 (8%)	0 (0%)
Iranian	0 (0%)	1 (10%)
**Education status, *n* (%)**		N/A
Bachelor's degree or higher	5 (38%)	
Diploma	4 (30%)	
TAFE certificate	3 (23%)	
Year 12 (HSC)	1 (8%)	
**Work status, *n* (%)**		N/A
Employed	7 (54%)	
Retired	3 (23%)	
Unemployed	2 (15%)	
Other (break)	1 (8%)	
**Back pain duration, median (range), years**	7 (0.2–50)	N/A
**Average pain intensity (0–10), median (range)**	6 (3–10)	N/A
**Geographic region in Australia, *n* (%)**	11 (85%)	9 (90%)
Metropolitan	2 (16%)	1 (10%)
Regional/Rural		
**Years of practicing, median (range)**	N/A	23.5 (6–31)

Four themes emerged among individuals with back pain, and three themes among GPs. The themes and supporting quotes are summarized in Tables 3 and Table 4.

**Table 3:** Themes and sub-themes for individuals with back pain.

Sub-themes	Quote number	Quote
**Theme: “I would happily, but there is not much out there”**		
Description: Participants’ awareness and experience with digital decision-support tools for back pain		
Decision aids currently used	Q1	P9: “So for my journey, I have always jumped on Google and done all my own research and from the minute that I actually started experiencing back pain, I started researching it.”
Decision aids currently used	Q2	P5: “I use Google, but not for the low back pain.”
Decision aids currently used	Q3	P12: “There's not much out there if I'm completely honest.”
Desire to use digital decision aids	Q4	P12: “If I could avoid going to the GP, I would happily try a digital tool.”
**Theme: Complementary support for patients and GPs**		
Description: Participants’ perspectives on how digital tools can support decision-making within the patient–clinician relationship		
Provide knowledge in conjunction with the consultation process	Q5	P4: “So it's probably more just trying to educate myself a little bit before I see the clinician. But then, as I said afterwards as well. So yeah, it's a bit of both.”
Current purpose of decision aid use	Q6	P12: “If I don't get an answer from the doctors, I tend to look up the extras that are adding on to the symptoms.”
Provide knowledge for condition management and treatment	Q7	P2: “But in terms of prevention and monitoring, when it comes to back pain or any other health condition, you've got to do self-directed research online, otherwise you'll be in a lot of trouble.”
Provide high-quality information to both patients and GPs	Q8	P9: “So if they did that, you know, if they did their degree 30 years back and they haven't updated themselves on low back pain, for example, digital decision aids would be really good because it would be putting updated information in their (GP's) hand.”
**Theme: “I would be hesitant”**		
Description: Participants’ attitudes toward safety and trust when using digital technologies		
Trustworthiness of digital decision aids	Q9	P5: “If you look it up on Google, Google gives you ideas from all over the world. You don't know which are good which are rubbish, which are fact, which are fiction. When it comes to the human body, I don't believe that there's a space for Google.”
Technology	Q10	P5: “Maybe I'm a little bit old for the digital world.”
Individualization	Q11	P9: “I personally would be very cautious to use like something that was developed by someone and then used by like every single specialist. Because I've found with back pain, it's so individualized to the person that if I was to use a specific tool, I don't think I would put very much weight on that being like right, and that's based on my experiences.”
Cost	Q12	P12: “If it cost straight up, no.”
GP factors	Q13	P5: “If I went to the doctor and he looked at me, examining me, and then he goes on and looks at Google to see what he's what he's doing, I wouldn't be very comfortable, quite honestly.” (within the context of confidence in the GP's decisions)
GP factors	Q14	P4: “That would be probably better because they can advise you on the information that's being provided to you on whether it's kosher. So I think it's probably good.”
**Theme: Integration into routine practice**		
Description: Participants’ reflections on how digital tools could be incorporated into everyday general practice.		
Accessibility of digital decision aids	Q15	P2: “I guess the thing is that some of us are more health literate and more information literate than others. So probably to address that it would be good to have more educational tools online that can actually help people sort through all the information that's online. Make sure they're looking at the right things, absorbing it the right way.”
Holistic nature of digital decision aids	Q16	P9: “Because if you were to make a good decision tool, I would want it to have more of a holistic input of information. So not just dealing with back pain from a medication standpoint or surgery standpoint, but also from an exercise standpoint also from stretching standpoint and also having it available to specialists or any person that someone would see if they were to have back pain.”
Digital decision aids can improve the consultation process	Q17	P6: “GPs unfortunately have very limited time on the consultation. If they are able to simplify the process by using a digital tool that's going to really help rather than just giving us palliative care.”

**Table 4:** Themes and sub-themes for GPs.

Sub-themes	Quote number	Quote
**Theme: “It certainly has its role”.**		
Description: Participants’ recognition of the value of digital tools in supporting back pain management.		
Limited experience with digital decision aids	Q18	GP2: “I've only been using pamphlets, paper type of aids rather than digital.”
Timing of use of the decision aid	Q19	GP1: “It would save time for sure, if they've already been through this checklist of things and had a look at it. And it comes up with something like, you know, an investigation in radiological investigations would be advisable or not advisable.”
Influence and acceptance of digital decision aids for patients	Q20	GP4: “If I was a patient, I would like to be able to have the option of some sort of digital decision aid because it might tell me yes, I need to seek medical advice urgently or less urgently, and it would improve my health literacy, which I think is important.”
The use and role of digital decision aids	Q21	GP6: “I don't think it would be a bad thing if they can check the boxes and show you what they've done when they come in. I think that would be that would be useful and save a bit of time and it might be a good way to sort of discuss it rather than them coming in and say I need an X-ray. And if they feel more involved in the whole process, I think it might be positive for both doctor and patient.”
Influence and acceptance of digital decision aids for patients	Q22	GP2: “I think, it depends on the patients. Not everyone would want to fill a survey before they come in or can go on to the computer or you know fill in some digital questions and all that. So, I feel it certainly has its role, but it may not be for everyone.”
**Theme: “I have concerns”**		
Description: Participants’ reservations about using digital technologies in healthcare		
Timing of use of the decision aid	Q23	GP3: (Discussion surrounding digital decision aids being used by patient after consultation) “I think it would be fraught with danger because it's almost like the patient will use it to check on us and unless the algorithm is extremely accurate, it would cause a rift between what we think is right versus what a computer or digital tool thinks is right.”
Need for consult	Q24	GP1: “So if someone's only relying on a decision-making tool which is purely related to the musculoskeletal system or the spinal system, then another medical professional could do that. It doesn't have to be your GP.”
Are digital decision aids required	Q25	GP10: “No, sorry. Doesn't work for me. I don't need it.”
GP instincts	Q26	GP3: “Sometimes as GP's. I hope all of you would agree that sometimes it's our gut feeling that, you know, we know the patient well. We've seen them for many years and something's different. Something not right about them, the way they're behaving or something about their social history is a bit odd. That won't be easily calculated on an algorithm, and we would then investigate further and often we pick things up that we can't easily explain, just on history.”
Limitations of digital decision aids	Q27	GP3: “I don't think I'll be using a clinical tool in this type of because our patients are undifferentiated when they come to us. They could have anything from, you know, we mentioned pancreatic cancer could be endometriosis.”
Patient–practitioner alliance	Q28	GP1: “There's more of a partnership (with the use of digital decision aids) and I think they'd go away feeling more supported and more comfortable with how the consults gone.”
Limitations of digital decision aids	Q29	GP8: “So yeah, I have a lot of concerns about that. Unless the source is very very solid, and they are directed to use a particular you know a particular website or a particular evidence-based source of information.”
**Theme: Integration into clinical practice**		
Description: Perspectives on how digital tools could be effectively integrated into clinical workflows to support decision-making		
Practitioner preference on interactions in consultations	Q30	GP3: “I tend to prefer to ask the questions myself. I think patients value the doctor being the one asking rather than being handed or being asked to complete a questionnaire. I think part of that consultation is to read the patient's body language, see how they react to questions. I think I still, despite the option of having (digital decision aids). I would prefer to do the question myself.”
Features of digital decision aids that influence usability	Q31	GP8: “I guess if they if they really, determine if they're easy to use. If they're part of the software program, I guess you know, and if it really did make a difference in terms of management of a patient, if you're if you want me to think broadly, if they say OK, well the MBS guidelines stipulate that you do get funding for imaging only if these criteria are met. That would make you definitely use decision aids a bit more, just like PBS criteria, which are stipulated in the software.”
Integration of digital decision aids	Q32	GP4: “Another thing for me is that, if this is going to be a decision aid that I am going to routinely use in the consult it has to be included natively in best practice which is the practice management software that I use because once you navigate outside of best practice and use it through a web browser. It's hard to document that it's been done.”
Value and perceived effectiveness of digital decision aids	Q33	GP5: “Then it will save us a good value. Basically, it will add a value to our consult plus time. Yeah, I think it's achievable to have an appropriate tool to measure and address this pain using these simple elements.”
Accessibility of digital decision aids	Q34	GP7: “Have some education to the public. These decision aids on these dates are available to everybody. And to look it up, fill it in before they come into the consultation and that can be applied to many diseases as well.”

### Perceptions of digital decision support tools among individuals with back pain

#### Theme: “I would happily, but there is not much out there.”

Individuals with back pain reported limited experience using *digital* decision support tools in general practice. Participants commonly used Google and webpages primarily to gain information about diagnosis and treatments for back pain. Those tools were used independently of GPs, as reported by a 24-year-old man (Q1). Two older participants in the study reported that they had not previously used digital materials to support their decision about back pain management (Q2). A 24-year-old participant explained that digital decision support tools are rarely used because few are available for back pain in general practice (Q3). However, most participants expressed a desire to use digital materials to support back pain management, primarily driven by unmet needs in the current practice (Q4).

#### Theme: Complementary support for patients and GPs

Participants noted that digital technologies could be beneficial before and after consultations to support discussions and decision-making (Q5). An 81-year-old male with a 50-year history of back pain suggested that educational materials could address persistent post-consultation questions (Q6). A 34-year-old female highlighted the value of complementary online searches for prevention and symptom monitoring (Q7). Similarly, a 32-year-old female noted that GPs could use credible online resources to update knowledge and improve care (Q8).

#### Theme: “I would be hesitant.”

Most participants were concerned about the trustworthiness of online resources and technologies in supporting health decisions, including their reliability, accuracy, and the credibility of the information sources, especially when used without a GP's endorsement. An 81-year-old male was skeptical about whether digital tools, such as Google, can reliably answer and explain health-related questions (Q9). The same participant recognized that his skepticism might be associated with his age and limited familiarity with digital technologies (Q10). A few participants questioned whether digital resources could personalize information for patients (Q11) and expressed concerns that the potential costs of digital technologies might limit access to such tools (Q12). The final challenge raised was participants’ discussions about GPs relying too heavily on digital technologies to make decisions for their patients. An 81-year-old male expressed that he would lose confidence in his GP if he were to use digital decision support tools during the consultation to check treatment options (Q13). However, GPs’ use of digital technologies was perceived as more trustworthy when it supported the interpretation of findings, confirmed results, and clarified information. A 54-year-old female stated that she would be comfortable if GPs used support tools to help interpret and verify or reinforce information during the consultation (Q14).

#### Theme: Integration into routine practice

Participants discussed the importance of incorporating digital materials to support decisions for back pain management into routine practice. A 54-year-old woman noted that embedding decision tools into routine care requires patients to possess digital and scientific literacy; without this, such tools may go unused or be used ineffectively (Q15). A 32-year-old woman suggested that providing details on pharmacological, surgical, and non-pharmacological options could motivate patients with back pain to continue using digital tools (Q16). Some participants indicated that integrating digital tools into consultations could help GPs organize and simplify treatment plans. For example, a 44-year-old woman noted that such tools could support self-management rather than temporary or symptom-based solutions (Q17).

### Perceptions of digital decision support tools among GPs

#### Theme: “It certainly has its role”.

GPs reported experience with non-digital decision support tools, such as printed education materials (i.e. fact sheets), to support patients in following treatment and lifestyle recommendations. They were largely unaware of specific *digital* decision-support resources available to help GPs and patients manage back pain in general practice (Q18). Most GPs considered that digital tools would be valuable for pre-consultation screening (Q19). A 31-year-old male GP noted that digital tools could assist patients in deciding whether to seek care and in preparing for consultations through enhanced health literacy (Q20). A 59-year-old male GP similarly noted that digital tools could help GPs screen for red flags before consultations, freeing up time for meaningful discussions with patients that commonly are not possible due to time constraints (Q21). However, a 54-year-old female GP stressed that not all patients would be willing to complete forms before the consultation (Q22).

#### Theme: “I have concerns”.

Many GPs expressed concerns about the impact of digital decision support tools on consultations and clinical interactions in the management of back pain. A few GPs felt uncomfortable with the possibility that patients might use the tools to check whether clinicians had provided the right approach. For example, a 43-year-old male GP mentioned that inconsistencies between the tool and clinicians’ recommendations could undermine the therapeutic relationship (Q23). A 51-year-old female GP expressed concern about the independent use of digital decision-support tools, suggesting that this could undermine GPs’ professional role (Q24).

A few GPs reported not requiring decision support tools for managing back pain, citing confidence in their existing knowledge and clinical skills (Q25, Q26). Others believed that current technology is not ready to address complex cases, particularly when patients present with multimorbidity or back pain due to uncommon causes (Q27). These concerns seemed to diminish when decision support tools were designed for patient use under GP supervision (Q28) and when the information provided was transparent, evidence-based, and trustworthy (Q29).

#### Theme: Integration into clinical practice

GPs discussed the importance of embedding digital decision-making tools within routine care to support effective implementation. Overall, GPs agreed that decision support tools should be seamlessly integrated into the existing general practice workflow and not be intended to replace clinical interaction (Q30). A few GPs suggested that digital tools designed to facilitate patients’ and GPs’ decisions should be integrated into existing practice management systems (e.g. pre-appointment platforms that already capture patient information) to support implementation in clinical settings (Q31/Q32). They further highlighted that these tools should be user-friendly, efficient, and add value to general practice—for example, by monitoring symptoms or reducing administrative workload (Q33). Finally, they emphasized the importance of informing the public on how to use these tools (Q34).

## Discussion

This study explored the perceptions of individuals with back pain and GPs regarding digital materials or tools accessed by patients or health professionals at any point in the consultation to support decision-making. Separate themes revealed complementary views to inform future implementation, with both groups often relying on generic tools. The discussion was structured to facilitate the research and implementation of digital materials across three stages of the clinical practice: before, during, and after the consultation.

### Before the consultation

Individuals with back pain reported using search engines such as Google to understand the cause of their back pain and gain information about treatment options before and after the consultation. However, most of them recognize uncertainty regarding the trustworthiness of information sources, which, according to a systematic review, more than half of websites provide inaccurate or unclear treatment recommendations [23]. This suggests that, despite their desire for reliable information about treatment options, patients often lack access and therefore turn to untrustworthy digital resources. Access to trustworthy digital materials could be encouraged by clinicians, practices, and health insurance providers.

In this study, we found that GPs, and especially individuals with back pain, believe that pre-consultation decision-support tools could increase patients’ knowledge and enable productive discussions. However, low evidence-based literacy and maladaptive pain beliefs may hinder patients’ use of digital tools and affect decision-making. These barriers can limit their understanding of treatment options and reduce their ability to engage in collaborative discussions with clinicians [24]. For some patients and professionals, education materials and time for assimilation may be required to support implementation.

Another facilitator is the integration of education materials and tools into primary care workflows, as consistently reported by both patients and GPs. For example, digital pre-consultation tools could provide an opportunity to educate and prepare patients for the consultation [25]. A recent randomized controlled trial demonstrated that a pre-appointment form, which facilitated the delivery of information about back pain management, improved patients’ perceptions of how well they were prepared to engage in discussions with their GPs and to make informed health decisions [26].

GPs indicated that tools to screen for red flags before the consultation would be valuable, as they could help validate clinical decisions and allow more time during the consultation to address patients’ questions and provide education. For instance, Traeger et al. [11] developed a tool to estimate the risk of chronic back pain (PICKUP; https://myback.neura.edu.au/), which incorporates red flags as indicators of prompt GP visits. Although the prognostic model requires further external validation and refinement, it has been suggested that it could prevent up to 40 unnecessary GP visits per 100 patients presenting to primary care [11] and could be easily implemented in pre-consultation tools for back pain. Advancements in digital technologies, such as artificial intelligence (AI), may facilitate the identification of urgent cases and enable timely advice to patients before or during a general practice visit [27]. For example, large language models can identify and analyze patients’ self-reported symptoms in electronic health records, flag potentially serious conditions, and provide suggestions for further investigations, self-care, or referral [28]. Nevertheless, testing is needed to evaluate their acceptability among patients and clinicians, the reliability of their outputs, and their feasibility for integration into routine care [12].

These pre-appointment strategies become especially relevant in the context of prolonged waiting times for accessing health services, when patients are at a greater risk of relying on inaccurate information or missing timely reassurance and evidence-based advice.

### During and after the consultation

Interestingly, GPs demonstrated confidence in making treatment decisions for back pain, even though evidence of non-guideline concordant care. A systematic review [6] found that GPs prescribed second-line interventions, such as NSAIDs, in 35% of acute and chronic back pain, compared with only 20% providing advice to stay active or referring to a physiotherapist, which are considered first-line options [29].

One possible explanation for this discrepancy may be the entrenched medication culture within general practice, as well as the lack of a standardized system for prescribing non-pharmacological interventions [30]. Recent initiatives have aimed to promote the prescription of non-drug treatments in general practice by increasing access to national clinical guidelines [5, 31] and high-quality systematic reviews and overviews [32]. For example, the *Cochrane Database of Systematic Reviews* has recently published a series of overviews on pharmacological, non-pharmacological, and non-surgical interventions for back pain [33, 34], which are accessible via the *Cochrane Library* [32]. Nonetheless, most evidence-based information resources have yet to be systematically integrated into general practice management systems. In Australia, the e-HANDI project (https://e-handi.com/), an online prescription platform, allows GPs to enter a health condition and generate evidence-based non-pharmacological options that can be emailed directly to patients, including information on benefits, contraindications, adverse effects, and practical details, such as frequency, dose, and community availability. Research on mobile app interventions for back pain is also promising, suggesting that GPs could prescribe evidence-based apps during consultations [35].

Co-design of future decision-making tools with patients, clinicians, and policymakers might increase acceptability, feasibility, and ultimately implementation. For example, interviews with individuals with back pain and health professionals were used to co-create a decision-support tool for lumbar fusion [36].

While promising, all these tools need to address challenges related to digital health literacy, age-associated barriers, internet access, information reliability, acceptability, and effectiveness [37]. Over time, the influence of these barriers may diminish as digital technologies become increasingly accessible through inclusivity initiatives [38]. Importantly, both patients and clinicians acknowledge the complementary role of digital technologies within GP-led care, emphasizing their potential as a “tool to support, not replace.”

### Limitations

A medical doctor (O.B.) is among the authors, and the study was discussed with GPs during the conceptualization. However, no actively practicing GP was involved. A more extensive member-checking process with participants could have further strengthened confidence in the findings. Future studies with more participants living/practicing in rural and remote areas, and culturally and linguistically diverse backgrounds can enhance transferability.

## Conclusion

Our study combined GP and patient perspectives to generate insights for implementing digital decision-support tools in primary care. We highlight their potential to support back pain management and the need to consider context, patient, and GP views for successful implementation.

## Supplementary Material

cmaf098_Supplementary_Data

## Data Availability

The data underlying this article will be shared on reasonable request to the corresponding author.
