# Supplementary material for: “A tool to support, not replace”: patient and general practitioner perceptions of digital decision support tools for back pain
Source: Fam Pract. 2025 Dec 31;43(1):cmaf098. doi: 10.1093/fampra/cmaf098 (PMC12754595; doi:10.1093/fampra/cmaf098)
Supplement: cmaf098_Supplementary_Data [file cmaf098_supplementary_data.zip › Supplementary data.pdf]

Table of Contents

CONSOLIDATED CRITERIA FOR REPORTING QUALITATIVE STUDIES (COREQ): 32-ITEM CHECKLIST 2

FOCUS GROUP GUIDE FOR PATIENTS. 7

FOCUS GROUP GUIDE FOR GENERAL PRACTITIONERS. 11

Consolidated criteria for reporting qualitative studies (COREQ): 32-item checklist

No. Item	Guide questions/description	Response/ Reported on Section
Domain 1: Research team and reflexivity		
Personal Characteristics		
1. Interviewer/facilitator	Which author/s conducted the interview or focus group?	First and last author<br>Data collection and analysis
2. Credentials	What were the researcher's credentials? E.g. PhD, MD	Researchers with experience in clinical practice, qualitative study, back pain, digital health, and honours students.<br>Reflexivity and Assumptions
3. Occupation	What was their occupation at the time of the study?	Researchers with experience in clinical practice, qualitative study, back pain, digital health, and honours students.<br>Reflexivity and Assumptions
4. Gender	Was the researcher male or female?	Moderators: males<br>Authorship: 2 females<br><br>Reflexivity and Assumptions
5. Experience and training	What experience or training did the researcher have?	PhD, Physiotherapy, Exercise physiology, Medicine, Psychology, or Qualitative research<br><br>Reflexivity and Assumptions

Relationship with participants		
6. Relationship established	Was a relationship established prior to study commencement?	No<br>Participants and Recruitment /First paragraph of the Results
7. Participant knowledge of the interviewer	What did the participants know about the researcher? e.g. personal goals, reasons for doing the research	Research goals, professional roles<br><br>Data collection and analysis; Interview guide (Appendix)
8. Interviewer characteristics	What characteristics were reported about the interviewer/facilitator? e.g. Bias, assumptions, reasons and interests in the research topic	Professional roles<br><br>Data collection and analysis
Domain 2: study design		
Theoretical framework		
9. Methodological orientation and Theory	What methodological orientation was stated to underpin the study? e.g. grounded theory, discourse analysis, ethnography, phenomenology, content analysis	Relativism and interpretivism orientations<br><br>Reflexivity and Assumptions
Participant selection		
10. Sampling	How were participants selected? e.g. purposive, convenience, consecutive, snowball	Purposive, and snowball<br>Participants and Recruitment (Table 1)
11. Method of approach	How were participants approached? e.g. face-to-face, telephone, mail, email	Individuals with back pain: social media advertisement, telephone<br><br>General practitioners: email from recruitment company and telephone from the research team.<br><br>Participants and Recruitment (Table 1)
12. Sample size	How many participants were in the study?	23 in total (13 individuals with

		back pain and 10 GPs)<br><br>Participants and Recruitment
13. Non-participation	How many people refused to participate or dropped out? Reasons?	50 individuals with back pain expressed interest. 37 were not included (reasons described in the manuscript)<br><br>First paragraph of the Results
Setting		
14. Setting of data collection	Where was the data collected? e.g. home, clinic, workplace	Participants home or GPs' office.<br><br>Participants and Recruitment
15. Presence of non-participants	Was anyone else present besides the participants and researchers?	No<br><br>Data collection and analysis
16. Description of sample	What are the important characteristics of the sample? e.g. demographic data, date	Results (Table 2)
Data collection		
17. Interview guide	Were questions, prompts, guides provided by the authors? Was it pilot tested?	Yes (interview guide)<br><br>Appendix
18. Repeat interviews	Were repeat interviews carried out? If yes, how many?	No (cross-section qualitative study)<br><br>Study design and ethics approval
19. Audio/visual recording	Did the research use audio or visual recording to collect the data?	Yes (audio and video - Teams)<br><br>Data collection and analysis
20. Field notes	Were field notes made during and/or after the interview or focus group?	Paper notes during the interview, and whiteboard notes after the interview

		Data collection and analysis
21. Duration	What was the duration of the interviews or focus group?	One hour.<br><br>Data collection and analysis
22. Data saturation	Was data saturation discussed?	Yes<br><br>Participants and Recruitment "…sufficient understanding of the phenomenon was achieved…"
23. Transcripts returned	Were transcripts returned to participants for comment and/or correction?	No<br><br>Limitation described
Domain 3: analysis and findings		
Data analysis		
24. Number of data coders	How many data coders coded the data?	Two<br><br>Data collection and analysis
25. Description of the coding tree	Did authors provide a description of the coding tree?	No
26. Derivation of themes	Were themes identified in advance or derived from the data?	From the data<br><br>Data collection and analysis (inductive approach)
27. Software	What software, if applicable, was used to manage the data?	NVivo<br><br>Data collection and analysis
28. Participant checking	Did participants provide feedback on the findings?	No<br><br>Limitation reported
Reporting		
29. Quotations presented	Were participant quotations presented to illustrate the themes/findings? Was each quotation identified? e.g. participant number	Yes<br><br>Results (Table 3 and Table 4)
30. Data and findings consistent	Was there consistency between the data presented and the findings?	Yes

		Results
31. Clarity of major themes	Were major themes clearly presented in the findings?	Yes<br><br>Results (Table 3 and Table 4)
32. Clarity of minor themes	Is there a description of diverse cases or discussion of minor themes?	Yes<br><br>Results

Focus group guide for patients.

General welcoming

Thank you so much for taking the time to participate in this study.

You all have unique experiences that are informative for our study. You all have experience with low back pain and may be interested in improving the management of your condition in healthcare. Does anyone have questions about the study before we get started?

[if participants have questions - use the PIS to answer the questions]
[if participants do not have questions - proceed]

Before we start, I want to introduce the focus group's ground rules. These rules will ensure we make the most out of this experience.

Introducing ground-rules

- Ensure your first and last name are visible on the Teams screen.
- The discussion should take approximately two hours but may go a little longer.
- Mobile phones should be switched off or silent, not on vibrate.
- If you need to go to the bathroom, just turn off your camera before leaving and return quietly.
- Try to talk to each other rather than just answering the moderator.
- There are no right and wrong answers. Feel free to disagree with each other and offer alternative viewpoints. However, if you do disagree, please do so in a respectful manner.
- Please try not to talk over the top of each other, as this makes transcription of the group almost impossible, and we want to hear your viewpoints.
- The moderator may interrupt the group if these ground rules are not being followed.
- If you experience any distress or discomfort at any time, please let a researcher know or feel free to turn the camera off or leave.

Focus group questions

Part 1 - Specific questions about digital decision aids for low back pain. (first 45 minutes)

We want to understand your thoughts about digital tools to support decision-making in the management of low back pain.

Digital-decision-making tools refer to any tool used in person or online to support/assist decision-making (e.g. treatment decisions, management options) in healthcare.

These tools may help clinicians or patients identify or communicate the diagnosis, prognosis, or management of health conditions at any stage of the care.

There are different types of decision aids; some include non-digital tools, and others use digital tools that patients or clinicians can use before, during, or after consultations.

Non-digital tools are pamphlets or booklets clinicians deliver before or after the consultation to inform treatment options.

Digital tools include using Google, Webpages, ChatGPT, and other specialised platforms to identify, inform, and discuss treatment options at any point of the consultation.

Patients and clinicians can also use these tools independently or together in the consultation.

For this study, we want to understand your thoughts about using digital decision aids to support decision-making in healthcare.

Knowledge:
Have you heard of or used any form of **decision aid**?

- Can you tell us when you used it? Or can you tell us what you heard about it?
- What decision aids have you used?
- Was it before, during, or after the consultation?
- Was that in a digital format? (If not, what would it be like to receive it in a digital form such as a smartphone, email, webpage, or any other technology)?

Beliefs about consequences/ Knowledge
How do you think **decision aids using digital technologies** could influence back pain management?
- Is there a role in using digital decision-making before, during, or after visiting a general practitioner, physiotherapist, or other health provider?

['Let's create a scenario here. Suppose you both booked a consultation with your GP. You (on the right) did not use any decision aid. And you (on the left) used a decision

aid. Would there be a difference in the consultation or how you manage your condition?]

Social/professional role and identity/ Intention
How would you feel using digital decision aids in a consultation with your general practitioner or other health professionals for your back pain?
- Would you feel it is useful/suitable/appropriate?
- Would you feel it is desirable?

Skills/ Knowledge/ Social/professional role and identity/ Beliefs about capabilities/ Optimism/ Beliefs about consequences/ Memory, attention, and decision processes/ Environmental context and resources/ Skills
What would be the potential problems or challenges of using decision aids before, during, or after a consultation with a general practitioner or other health professionals for back pain? (e.g., patient, clinicians, practice, health insurance).
- Can you describe a situation in which decision aids could be problematic or challenging?

["Can we create together a scenario where a decision aid would be challenging or problematic?' You are the patient, and you are the clinician...].
Reinforcement/ Intentions/ Skills/ Knowledge/ Social/professional role and identity/ Beliefs about capabilities/ Optimism/ Beliefs about consequences/ Memory, attention, and decision processes/ Environmental context and resources/ Skills
What could be done to prevent these problems/challenges?
- What would help you use decision-making digital tools before, during or after a consultation for back pain?
- What aspects of the healthcare environment (e.g., patient, clinicians, practice, health insurance) would facilitate the use of decision-making digital tools?
["Can we create together a scenario where we do something to prevent or deal with the challenges of using a decision aid in primary care?' Let's create the challenging scenario again first... You are the patient, and you are the clinician... What would we do to make a decision aid work here?]
Summary
Final Question:
As we conclude the first focus group, is there anything else you want to note on digital decision aid for back pain?

Thank you for sharing your views. Does anyone have questions? We completed the focus group now.

Have a great day!

Types of probes for the interview:

- Silent: Give people the opportunity to express their opinions.
- Echo: repeat the last statement and ask the respondent to continue
- Neutral: encouraging; “I see” or uh-huh”
- Direct: “Tell me more”

- Phased assertion: implies we already know something or encourages the respondent.
- Detail: Who, where, what, when, how
- Clarifying: you said “x”, please describe what you mean by that
- If patient asks a question: “I will be happy to answer that after the interview...”
- Focus on topic and time limit.
- Leave enough time to answers questions.
- Avoiding interrupting the informant.
- Ask opinions for people who did not interact

Focus group guide for general practitioners.

General welcoming

Thank you so much for taking the time to participate in this study.

You all have unique experiences that may be extremely informative for our study. You all have experience with back pain management and may have been interested in knowing what can be done to improve the management of low back pain for patients in primary care. Has everyone had the chance to read what the study is about? Does anyone have questions?

[if participants have questions - use the PIS to answer the questions]
[if participants do not have questions - proceed]

Before we start, I want to introduce the focus group's ground rules. These rules will ensure we make the most out of this experience.

Introducing ground-rules

- Ensure your first and last name are visible on the Teams screen.
- The discussion should take approximately one hour but may go a little longer.
- Mobile phones should be switched off or silent, not on vibrate.
- If you need to go to the bathroom, just turn off your camera before leaving and return quietly.
- Try to talk to each other rather than just answering the moderator.
- There are no right and wrong answers. Feel free to disagree with each other and offer alternative viewpoints. However, if you do disagree, please do so in a respectful manner.
- Please try not to talk over the top of each other, as this makes transcription of the group almost impossible, and we want to hear your viewpoints.
- The moderator may interrupt the group if these ground rules are not being followed.

Focus group questions.

Specific questions about digital decision aids for low back pain

We want to understand your thoughts about decision aids to support decision-making in primary care.

Clinical decision aids refer to any tool used in person or online to support decision-making in primary care.

These tools may help clinicians or patients identify or communicate the diagnosis, prognosis, or management of health conditions at any stage of the care.

There are different types of decision aids; some include non-digital tools, and others use digital tools that patients or clinicians can use before, during, or after consultations.

Non-digital tools are pamphlets or booklets clinicians deliver before or after the consultation to inform treatment options.

Digital tools include using Google, Webpages, ChatGPT, and other specialised platforms to identify, inform, and discuss treatment options at any point of the consultation.

Patients and clinicians can also use these tools independently or together in the consultation.

For this study, we want to understand your thoughts about using decision aids to support decision-making in primary care.

Knowledge:
Have you heard of or used any form of **decision aid** for or with patients?

- Can you tell us the context of when you used the decision aid? Or can you tell us what you heard about it?
- What decision aids have you used or seen?
- Was it before, during, or after the consultation?
- Was that in a digital format? (If not, what would it be like if a patient used a decision aid in a digital format such as smartphone, email, webpage, or any other technology)?

Beliefs about consequences/ Knowledge
How do you think **decision aids using digital technologies** could influence back pain management?

- Is there a role in patients using digital decision-making before, during, or after visiting a healthcare provider?
- Is there a role in general practitioners using digital decision-making before, during, or after visiting a healthcare provider?

Social/professional role and identity/ Intention

How would you feel if patients used digital decision aids in a consultation with yourself for back pain?

- Why do you feel that way?

- Would you feel it is useful/suitable/appropriate?
- Would you feel it is desirable?

Skills/ Knowledge/ Social/professional role and identity/ Beliefs about capabilities/ Optimism/ Beliefs about consequences/ Memory, attention, and decision processes/ Environmental context and resources/ Skills
What would be the problems or challenges of using decision aids before, during, or after a consultation for a patient or practitioner? (e.g., patient, clinicians, practice, health insurance).

- Can you describe a situation in which decision aids could be problematic or challenging?

["Can we create together a scenario where a decision aid would be challenging or problematic?' You are the patient, and you are the clinician...].

Reinforcement/ Intentions/ Skills/ Knowledge/ Social/professional role and identity/ Beliefs about capabilities/ Optimism/ Beliefs about consequences/ Memory, attention, and decision processes/ Environmental context and resources/ Skills
What could be done to prevent these problems/challenges?

- What would help you or your patients to use decision-making digital tools before, during or after a consultation for back pain?
- What aspects of the clinical environment (e.g., patient, clinicians, practice, health insurance) would facilitate the use of decision-making digital tools?
["Can we create together a scenario where we do something to prevent or deal with the challenges of using a decision aid in primary care?' Let's create the challenging scenario again first... You are the patient, and you are the clinician... What would we do to make a decision aid work here?].
Summary
Final Question
As we conclude the focus group, is there anything else you want to note on digital decision aid for low back pain?

Thank you for sharing your views. Does anyone have questions? We completed the focus group now.

Have a great day!

Types of probes for the interview:

- Silent: Give people the opportunity to express their opinions.
- Echo: repeat the last statement and ask the respondent to continue
- Neutral: encouraging; “I see” or uh-huh”

- Direct: “Tell me more”
- Phased assertion: implies we already know something or encourages the respondent.
- Detail: Who, where, what, when, how
- Clarifying: you said “x”, please describe what you mean by that
- If patient asks a question: “I will be happy to answer that after the interview...”
- Focus on topic and time limit.
- Leave enough time to answers questions.
- Avoiding interrupting the informant.
- Ask opinions for people who did not interact.
